# Assessing diagnostic radiology knowledge among Syrian medical undergraduates

**DOI:** 10.1186/s13244-020-00937-9

**Published:** 2020-11-23

**Authors:** Mhd Obai Alchallah, Hlma Ismail, Tala Dia, Mosa Shibani, Mhd Amin Alzabibi, Fatema Mohsen, Khaled Turkmani, Bisher Sawaf

**Affiliations:** 1 Department of Internal Medicine, Faculty of Medicine, Syrian Private University, Mazzeh Street, P.O. Box 36822, Damascus, Syrian Arab Republic; 2 AL Kalamoon General Hospital, Ministry of Health, Damascus, Syrian Arab Republic; 3 Faculty of Medicine, American University of Beirut Medical Center, Beirut, Lebanon; 4 Internal Medicine Department, Hamad General Hospital, Hamad Medical Corporation, Doha, Qatar

**Keywords:** Radiology, Awareness, Knowledge, Syria, Medical students

## Abstract

**Background:**

The role of radiology in medicine and healthcare is rapidly expanding worldwide, but awareness about this field among medical students is poor. This is the first study to assess Syrian medical students’ knowledge and attitude regarding radiology.

**Methods:**

This is a cross-sectional study conducted at the Syrian Private University, on November 8, 2019, on the International Day of Radiology during the war crisis. Data were collected through self-administered surveys and analyzed using the Statistical Package for Social Sciences version 25.0 (SPSS Inc., Chicago, IL, USA).

**Results:**

The questionnaire was completed by 269 students whose ages ranged between 17 and 30 years old. Males constituted 63.6% of the respondents. The results revealed adequate knowledge about the basics of radiology. 73.6% of the students had previously heard about interventional radiology. There were slight misconceptions with certain points in each section, especially those pertaining to the radiation exposure of each imaging method. Finally, the students expressed low interest in radiology as a future career (24.5%).

**Conclusions:**

The level of awareness can affect a student’s decision in considering radiology as a future career. Further evaluation of the methods of teaching, input from medical boards, curriculum advisors, and guidance from radiologists is required.

## Introduction

Medical imaging is any technological process used to view and create data about the human body for diagnosing, monitoring, or treating a medical condition [1]. There are many types of technologies, such as computed tomography (CT), magnetic resonance imaging (MRI), X-ray radiography, ultrasonography (US), and positron emission tomography (PET), which offer different information depending on the body part on which they are used [1]. Therefore, imaging is a critical and growing component of modern medical diagnosis and practice. Its importance lies in its ability to diagnose and reduce unnecessary procedures safely and effectively [2]. When imaging is used for diagnostic purposes, it is termed diagnostic radiology. In recent years, technological innovations have given rise to the field of interventional radiology (IR) which uses imaging for the treatment of various diseases [3]. What is surprising is how little is done to promote awareness about diagnostic/interventional radiology during the early stages of medical education [3]. Students should have sufficient knowledge about radiation, as it is a vital part of their future medical practice.

Imaging has been utilized at twice the rate of other healthcare technologies, which is suggestive of inappropriate applications of imaging [4]. Imaging-associated radiation increases the risk of cancer even at low-dose exposure [5]. In Australia, the cumulative risk of cancer, up to the age of 75 years, that is attributed to diagnostic imaging is around 1.3% (430 cases per year) [6]. A big part of imaging overutilization is due to gaps in physicians’ knowledge regarding imaging safety and indications. Studies have shown that physicians’ lack of expertise in determining which radiological tests are most appropriate is subjecting the patients to unnecessary interventions [4, 7, 8]. Thus, educating physicians on appropriate utilization, safety, protection, and risks of radiology is crucial for optimal patient care [9, 10].

In 2018, Sawaf et al. [11] conducted a study on the specialty preferences of Syrian medical students, and they showed that less than 5% were interested in radiology in Syria. Medical students rarely consider radiology as a career path for reasons such as the scarcity of postgraduate (PG) courses [12–15], and the insufficiency of knowledge offered on ionizing radiation and radiation protection during the preclinical years and the clinical training period [16, 17]. In 2004, the Pan Arab Association of Radiological Societies (PAARS) issued a report that revealed a shortage in the number of radiologists in Syria (400 Radiologists in Syria with a ratio of 24 Radiologists/10^6^ Inhabitants) [18]. Many studies have also shown that medical students know little about dosage and associated risks that come with the utilization of radiological imaging [12–15].

The ongoing Syrian crisis (2011–present) has had serious effects on all aspects of life in the country. Sadly, the continuing conflict and the waves of displacement have placed a massive strain on the healthcare system, where thousands healthcare workers (HCWs) have either died while fulfilling their duties, while others have fled the country. Healthcare facilities in Syria were attacked 139 times in 2018, accounting for the second-highest number of attacks worldwide [19]. As result, the number of available radiology facilities and radiologists in Syria is decreasing at a fast pace which is threatening the continuity of this vital sector.

To date, there has been no published evidence on radiology knowledge among Syrian medical students. This study assesses the knowledge and attitude of undergraduate Syrian medical students regarding diagnostic and interventional radiology. Our purpose is to study whether medical students at the Syrian Private University (SPU) have the general knowledge needed in radiology. A secondary aim is to determine whether their knowledge increases as they progress in their studies and engage in clinical practice. Finally, we aim to assess the level of interest of these students in radiology as a future career.

## Methods

### Study design, setting, and participants

We conducted a cross-sectional study using a convenience sampling method at the faculty of medicine, Syrian Private University (Syria) in Damascus during the International Day of Radiology, which took place on November, 8, 2019. All included participants were Syrian undergraduate medical students. Students were informed that their participation was voluntary and their anonymity was assured. We obtained ethical approval from the Institutional Review Board (IRB), Faculty of Medicine, Syrian Private University. We used a structured self-administered English questionnaire that was designed from several existing published studies [15, 20, 21]. Students were allowed to opt-out of the study at any given time while ensuring them that such a step would not affect their grades. The questionnaire contained 39 questions divided into seven sections. The first section consisted of 7 socio-demographic questions including: age, marital status, gender, current residence, educational year, GPA, and mother’s education level. The other six sections were divided into background and experience (8 questions); basic knowledge (9 questions); radiology as a screening test (4 questions); levels of radiation exposure (5 questions); and radiology as a career (6 questions). In the radiology knowledge sections, one point was given to each correct answer. Individual knowledge scores were calculated as the percentage of points obtained by each student, with 100% considered equivalent to the maximum number of points that a student can obtain. Mean knowledge scores were calculated as average of the individual scores. The questionnaire is available in Additional file 1: Appendix 1.

### Statistical analysis

We analyzed data using the Statistical Package for Social Sciences version 25.0 (SPSS Inc., Chicago, IL, United States). We reported categorical data as frequencies and percentages (for categorical variables), and continuous data as means and standard deviations (SD). Comparison of knowledge scores between two categories was carried out using unpaired Student’s *t* test or Mann–Whitney U test. Association between categorical groups was evaluated using Pearson Chi-square test. *p* value < 0.05 was considered statistically significant.

## Results

### Demographic characteristics

Out of 300 medical students, 269 filled the questionnaire, of which 171 (63.6%) were males and 98 (36.4%) were females. The age of the respondents ranged from 17 to 30 years with the majority being around 22 years old (21.61 ± 3.58 years). Second-year students represented a minority 9 (3.3%), while the majority of participants were fifth-year students (*n* = 87 (32.3%)). Finally, 244 students (90.7%) lived in Damascus, and 238 (88.5%) were single (Table 1).

**Table 1:** Socio-demographic characteristics: (*n* = 269)

Age	Under 20	62 (23%)	Mother’s education	Primary	20 (7.4%)
	20–25	193 (71.7%)		Secondary	21 (7.8%)
	Above 25	14 (5.2%)		High School	81 (30.1%)
Gender	Male	171 (63.6%)		University	114 (42.4%)
	Female	98 (36.4%)		Graduate	38 (12%)
Social status	Single	238 (88.5%)	Current residence	Urban	244 (90.7%)
	In a relationship	14 (8.9%)		Rural	25 (9.3%)
	Married	7 (2.6%)	College year	1st	31 (11.5%)
GPA	< 2.0	30 (12.4%)		2nd	9 (3.3%)
	2.0–2.5	147 (61.0%)		3rd	34 (12.6%)
	2.5–3.0	68 (19.9%)		4th	42 (15.6%)
	> 3.0	16 (6.6%)		5th	87 (32.3%)
				6th	66 (24.5%)

### Background and experience in radiology

Out of the 269 participants, 221 (82.2%) had had a radiograph at one time in their life, and 263 (97.8%) had relatives who did. Most students were interested in learning more about radiology (*n* = 250 (92.9%)), 72 (26.8%) had completed a clinical rotation in radiology, and 71 (26.4%) had never t heard about interventional radiology. The students rated their knowledge in radiology compared to other fields as poor (*n* = 51 (19.0%)), adequate (*n* = 117 (43.5%)), good (*n* = 91 (33.8%)), and excellent (*n* = 10 (3.7%)). Moreover, they classified their knowledge about radiation doses as poor (*n* = 77 (28.6%)), adequate (*n* = 114 (42.4%)), good (*n* = 68 (25.3%)) and excellent (*n* = 10 (3.7%)) (Table 2).

**Table 2:** Background and experience *n* (269)

	Yes	No
Have you had a radiograph (of any kind) before?	221 (82.2%)	48 (17.8%)
Has any of your relatives had a radiograph (of any kind) before?	263 (97.8%)	6 (2.2%)
Are you interested in learning more about this field?	250 (92.9%)	19 (7.1%)
Have you completed a rotation in radiology?	72 (26.8%)	197 (73.2%)
Have you ever heard about interventional radiology/before?	198 (73.6%)	71 (26.4%)

	Poor	Adequate	Good	Excellent
How would you rate your knowledge of radiology compared to other fields?	51 (19.0%)	117 (43.5%)	91 (33.8%)	10 (3.7%)
How would you rate your knowledge about the radiation dose of common radiological investigations?	77 (28.6%)	114 (42.4%)	68 (25.3%)	10 (3.7%)

**Fig. 1:**
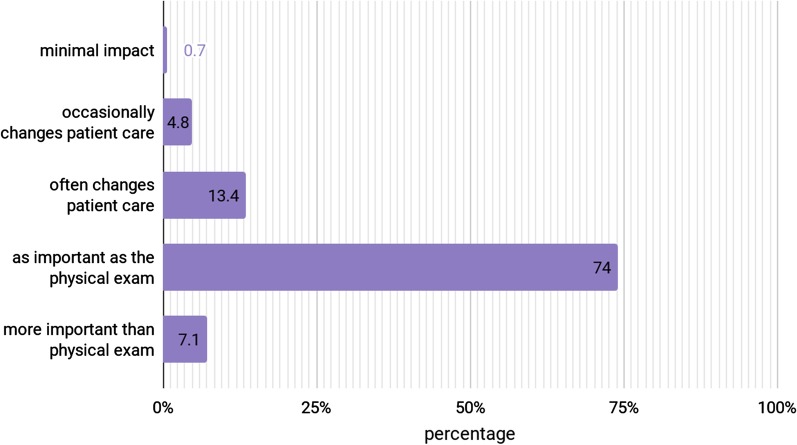
How much of an impact does radiology have on the diagnosis process?

The majority of participants (*n* = 199 (74%)) believed that radiology is as important as physical examination, and 36 students (13.4%) thought that radiology often changes patient care. On the other hand, 19 students (7.1%) believed that radiology is more important than a physical exam and 13 students (4.8%) thought that radiology occasionally changes patient care (Fig. 1).

### Knowledge about radiology

#### Radiology knowledge scores

The mean knowledge score of the sample was 49.2 ± 13.16% with the highest score being 87.50% and the lowest score being 16.67%. Students who completed a clinical rotation in radiology had an mean knowledge score of 51.85 ± 12.74%, and those who did not had an mean knowledge score of 48.22 ± 13.21% (Table 3).

**Table 3:** Mean knowledge scores of the participating medical students

	Knowledge score (%)		*t*	*p* value
	Mean	SD		
Total sample (*n* = 269)	49.2	13.16	N/A	N/A
Gender				
Male	49.34	13.65	8334.500	0.942
Female	48.94	12.32		
Completion of radiology rotation				
Yes	51.85	12.74	− 2.013	0.045
No	48.22	13.21		

#### Knowledge in radiology basics

The majority of participants (*n* = 214 (79.6%)) knew that children are the most sensitive age group to radiation, and 113 students (42.0%) knew that US is the safest radiological investigation. On the other hand, only 58 students (21.6%) knew that there is no specific limit to the number of radiographs that can be requested for a patient per year. There was a misconception about the imaging technique that is most harmful to the fetus as 107 students (39.8%) thought it was X-ray, while only 94 students (34.9%) correctly identified it as CT imaging. When asked about the most sensitive organs to radiation exposure, the students named the testis and ovaries (*n* = 175 (65.1%)), lungs and colon (*n* = 32 (11.9%)), liver, bladder and kidney (*n* = 24 (8.9%)), and breasts (*n* = 10 (3.7%)). Regarding CT contraindications, the answers were allergy to radio-contrast agents (*n* = 176 (65.4%)), pregnancy (*n* = 153 (56.9%)), renal failure (*n* = 115 (42.8%)), and liver failure (*n* = 38 (14.1%)). Finally, the students identified metal foreign bodies (*n* = 196 (76.9%)), pacemaker (*n* = 126 (46.8%)), and claustrophobia (*n* = 117 (34.5%)) as MRI contraindications (Table 4).

**Table 4:** Basic knowledge about radiology

What age group is the most sensitive to radiation?	Children	Teens	Adults	Elders	Others
	214 (79.6%)	13 (4.8%)	4 (1.5%)	18 (6.7%)	20 (7.4%)
Is there a specific number of radiographs that can be requested for the patient per year?	Yes	No			
	211 (78.4%)	58 (21.6%)			
Does radiation affect the fetus?	262 (97.4%)	7 (2.6%)			
What type most affects the fetus?	US	MRI	X-ray	CT	Others
	7 (2.6%)	52 (19.3%)	107 (39.8%)	94 (34.9%)	9 (3.3%)
What is the safest radiological investigation?	113 (42.0%)	35 (13.0%)	79 (29.4%)	25 (9.3%)	17 (6.3%)
What are the most sensitive organs to radiation?	Liver, bladder, kidney	Lungs, colon	Breast	Testis and ovaries	Others
	24 (8.9%)	32 (11.9%)	10 (3.7%)	175 (65.1%)	28 (10.4%)
CT contraindications:	Allergy to radio-contrast agent	Renal failure	Liver failure	Pregnant women	Do not know
	176 (65.4%)	115 (42.8%)	38 (14.1%)	153 (56.9%)	18 (6.7%)
MRI contraindications	Pacemaker	Metal foreign bodies	Claustrophobia	Do not know	
	126 (46.8%)	196 (76.9%)	117 (43.5%)	24 (8.9%)	

#### Radiation exposure

**Fig. 2:**
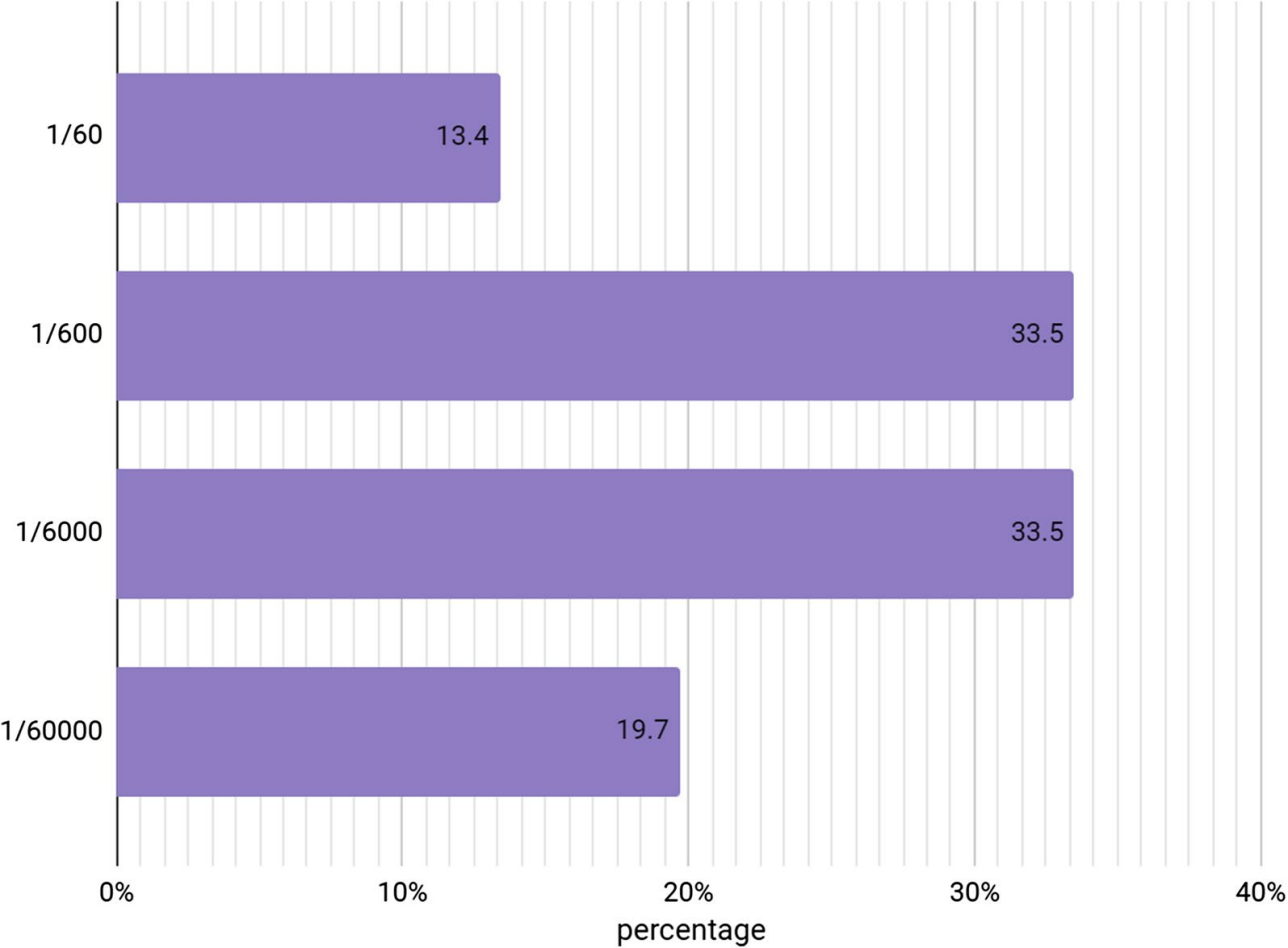
What is the best estimate that a 30-year-old women who undergoes a CT study of the abdomen and pelvis will develop cancer at some point in her life as a direct result of that study?

Regarding ionizing radiation exposure levels, only 45 students (16.7%) were aware that a chest CT is equivalent to 100–500 chest X-rays. Most students had misconceptions about MRI, as 194 (72.1%) of them believed that it uses ionizing radiation. On the other hand, 138 students (51.3%) knew that US does not employ ionizing radiation (Table 5). When asked to estimate the risk of a 30-year-old woman developing cancer after a CT study, the majority of answers were 1/600 (*n* = 90 (33.5%)) and 1/6000 (*n* = 90 (33.5%)). Other students incorrectly placed the risk at 1/60,000 (*n* = 53 (19.7%)) and 1/60 (*n* = 36 (13.4%)) (Fig. 2).

**Table 5:** Levels of radiation exposure: (*n* = 269)

Procedure	Number of units equivalent to a chest X-ray (a chest X-ray = 1 unit)					
	0	1–10	10–50	50–100	100–500	> 500
CT chest	33 (18.3%)	82 (30.5%)	46 (17.1%)	45 (16.7%)	*45 (16.7%)*	18 (6.7%)
MRI pelvis	*75 (27.9%)*	35 (13.0%)	57 (21.2%)	51 (19.0%)	34 (12.6%)	17 (6.3%)
PET-CT full body	14 (5.2%)	29 (10.8%)	35 (13.0%)	59 (21.9%)	61 (22.7%)	*71 (26.4%)*
US abdomen	*138 (51.3%)*	44 (16.4%)	28 (10.4%)	22 (8.2%)	26 (9.7%)	11 (4.1%)

Italics indicate the correct answer

#### Radiology as a screening test

Students showed good awareness about radiology as a screening tool, as they correctly identified mammography for breast cancer (*n* = 262 (97.4%)), US for abdominal aortic aneurysm (*n* = 186 (69.1%)), CT for lung cancer (*n* = 206 (76.6%)), and DEXA for osteoporosis (*n* = 212 (78.8%)) (Table 6).

**Table 6:** Radiology as a screening test

	Yes	No
We can perform a radiological screening test in (mammography for breast cancer)	262 (97.4%)	7 (2.6%)
We can perform a radiological screening test in (US for abdominal aortic aneurysm)	186 (69.1%)	83 (30.9%)
We can perform a radiological screening test in (CT for lung cancer)	206 (76.6%)	63 (23.4%)
We can perform a radiological screening test in (DEXA for osteoporosis)	212 (78.8%)	57 (21.2%)

#### Sources of radiology information

**Fig. 3:**
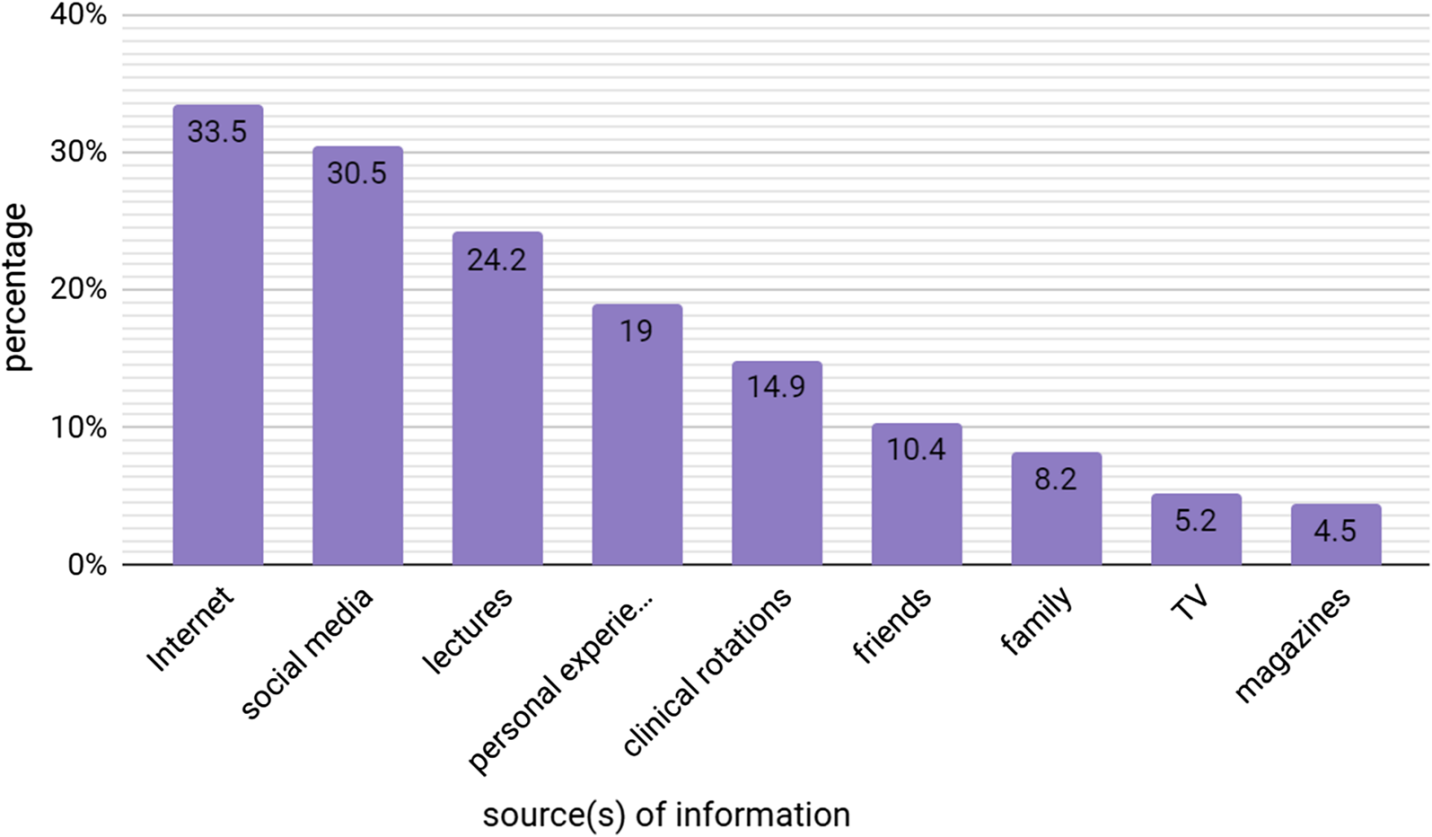
Source(s) of information on radiology

The majority of students relied on the Internet for information on radiology (*n* = 90 (33.5%)) followed by social media (*n* = 82 (30.5%)) and lectures (*n* = 65 (24.2%)). On the other hand, only 40 students (14.9%) learned about radiology during clinical rotations (Fig. 3).

### Radiology as a future career

**Fig. 4:**
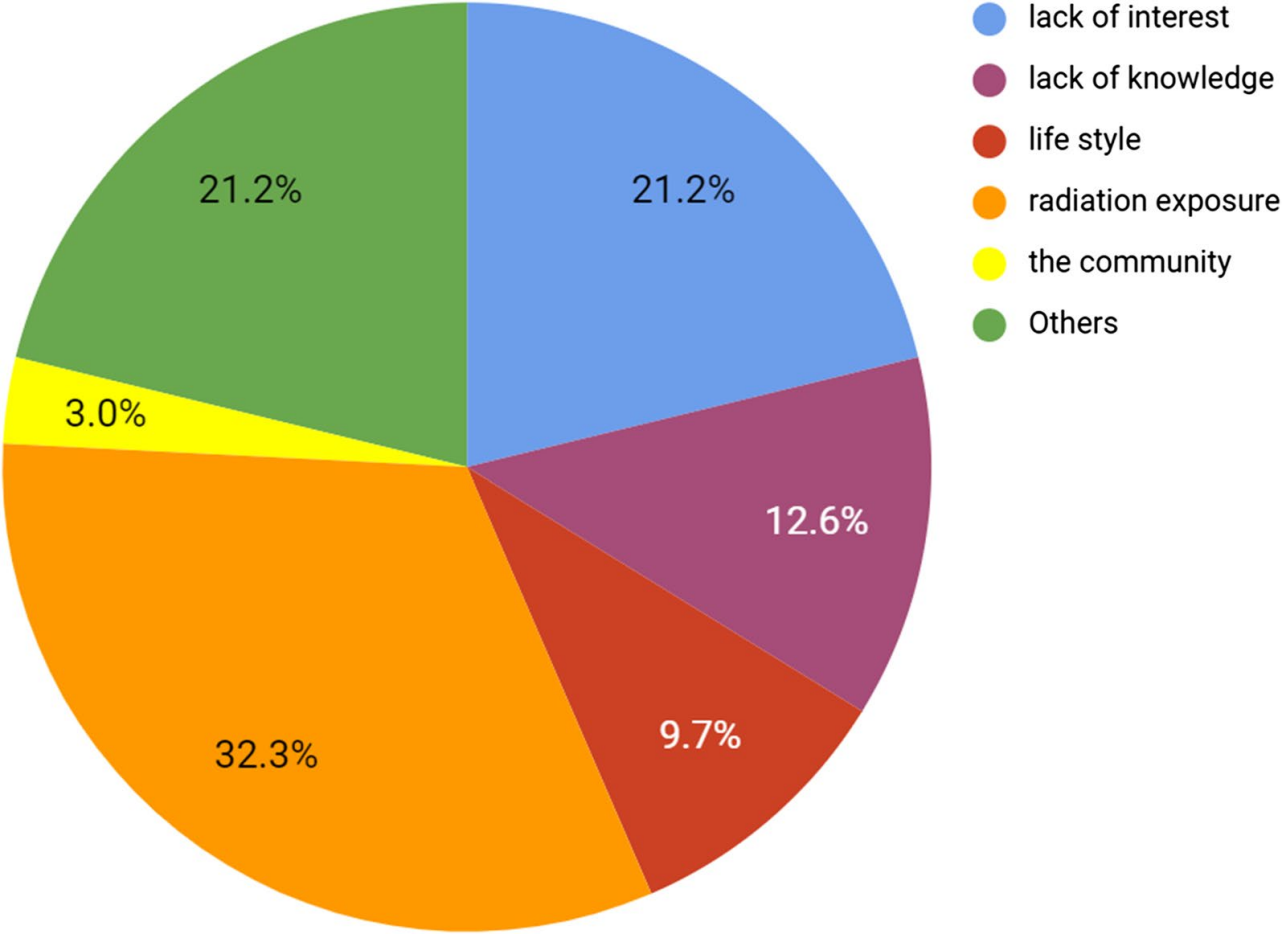
Why not? (Would you consider radiology specialty as a future career?)

A little over a half of the students (*n* = 140 (52.0%)) considered radiology to be interesting only when it relates to other fields of medicine, 61 students (22.7%) believed that radiology is dull but important, and only 52 students (19.3%) regarded radiology as interesting on its own. Moreover, about one-third of the participants (*n* = 89 (33.1%)) thought that radiologists have the same income as other specialties, and 101(37.5%) believed that the radiologist’s lifestyle is slightly easier than other specialties. Finally, a minority of medical students (*n* = 66 (24.5%)) considered radiology as a future career (Table 7). The most cited reasons for dismissing radiology as a career choice were fears of radiation exposure (*n* = 87 (32.3%)), lack of interest (*n* = 57 (21.2%)), and lack of knowledge (*n* = 34 (12.6%)) (Fig. 4).

**Table 7:** Radiology as a future career: (*n* = 269)

How interesting is the subject matter in radiology?	It is worthless to me	It is dull but important	It is interesting only as it relates to other fields of medicine	It is interesting in its own	
	16 (5.9%)	61 (22.7%)	140 (52.0%)	52 (19.3%)	
In your opinion, in general, a radiologist’s income for their services is compared with clinicians in medical and surgical specialties?	Much less	A little less	The same	A little more	Much more
	27 (10.0%)	74 (27.5%)	89 (33.1%)	46 (17.1%)	33 (12.3%)
How many years is radiology residency?	3	4	5	6	
	45 (16.7%)	153 (56.9%)	68 (25.3%)	3 (1.1%)	
In your opinion, in general, a radiologist’s professional lifestyle is compared with clinicians in medical and surgical specialties?	Much easier	Slightly more easier	The same	Slightly more difficult	Much more difficult
	59 (29.9%)	101 (37.5%)	68 (25.3%)	28 (10.4)	13 (4.8%)
Would you consider a radiology specialty as a future career?	Yes	No			
	66 (24.5%)	203 (75.5%)

### Comparative studies

**Fig. 5:**
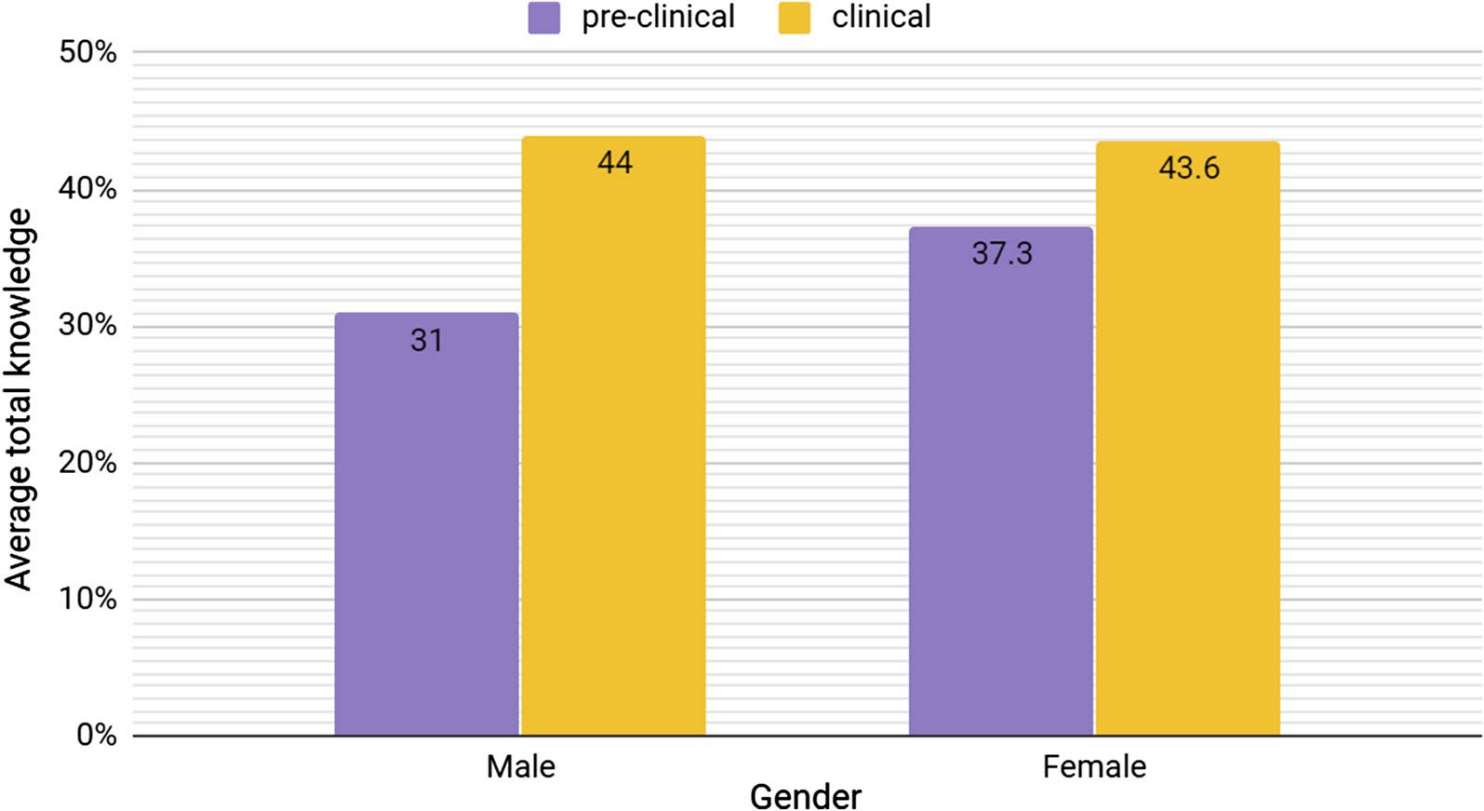
Comparison between clinical and preclinical years knowledge in both genders

We compared the knowledge scores of males and females and found no remarkable difference (Mann–Whitney U = 8334.500, *p* = 0.942). Interestingly, we only observed a slightly significant difference between the knowledge scores of the students who completed a rotation in radiology and those who did not (Mann–Whitney U = 8334.500, *p* = 0.942) (Table 3). Moreover, the students who rated their knowledge in radiology as good/excellent were found to be significantly more knowledgeable than those who described their knowledge as poor/adequate (*t* = 2.543, *p* = 0.012) (Table 8). The findings indicated a clear difference between clinical and preclinical students’ awareness especially among males in favor of the clinical students (Fig. 5). Finally, the results revealed a significant association between awareness about IR and attitude toward radiology specialty as a future career, with the majority of students who knew what IR is considering specializing in radiology in the future (*n* = 52 (78.8%)) (*χ*^2^ = 21.879, *p* = 0.000) (Table 9).

**Table 8:** The relation between self-evaluation and actual total knowledge

Group statistics					*T* test		
		*N*	Mean	Std	Mean difference	*T* test value	*p* value
Average total knowledge							
Good/excellent		101	44	15.7	− 4.8	2.534	0.012*
Poor/adequate		168	39.2	14.6			

*Statistically significant results (*p* value < 0.5)

**Table 9:** The relation between perception of IR and considering radiology as a specialty in future

	Have you ever heard about (interventional radiology)? (Yes)			Chi-square test	
		*N*	%	Chi-square value	*p* value
Would you consider a radiology specialty as a future career?	Yes	52	78.8%	21.879	0.000*

*Statistically significant results (*p* value < 0.5)

## Discussion

Our study assessed the knowledge of 269 Syrian medical students in radiology and their attitude toward this specialty as a future career. Our results showed comparable knowledge scores between males and females with no significant difference. Similar findings were reported by Alnajjar et al. [3] who found that awareness about interventional radiology among Saudi medical students was gender-independent. Furthermore, our students demonstrated adequate knowledge in the basics of radiology and radiation exposure. However, the majority (62.5%) rated their knowledge as poor/adequate, while only 37.5% felt they have good/excellent knowledge in radiology. Interestingly, our observations were very similar to those made by Leong et al. [22] who found that 66% of final-year medical students in a European country rated their knowledge in IR as poor/no knowledge, while only 33.4% thought they had adequate/good knowledge. Finally, our results indicated that students who rated their knowledge as good/excellent achieved a higher knowledge score than those who thought they had adequate/poor knowledge in radiology. Increasing evidence is emerging on the correlation between the level of confidence of medical students about their information in radiology and the actual knowledge they demonstrate in this field. O’Sullivan et al. investigated this topic in their study of medical students’ awareness of radiation exposures associated with diagnostic imaging investigations. They revealed that medical students who described their knowledge in radiology as excellent/good achieved a mean knowledge score of 76%, while students who felt they had adequate/poor/no knowledge in radiology had a much lower mean knowledge score of 52.4% [23].

The majority of our participants (79.6%) correctly identified children as the age group most affected by radiation. Our observation was in agreement with other studies. O’Sullivan el al. [23] indicated that 80% of their participants selected children as the most sensitive group to ionizing radiation. Kada [20], who studied the knowledge of radiation dose and risks among Norwegian final-year medical students, found that 94% of the students correctly identified children as the most susceptible group to radiation risks. Taken together, these results show that raising awareness on the risks of radiation is in the core of medical education programs worldwide. Furthermore, almost all students in our study (97.4%) were aware that radiation affects the fetus. However, there was a notable misconception regarding the most harmful type of imaging to the fetus, as 39.8% of the students indicated it was X-ray, while only 34.9% correctly identified it as CT. It should be noted that since ethics prohibit experimentation on human fetuses, there are few reports on the dangers of imaging-associated radiation on the fetus. The only information available comes from the observations of patients who survived Japan’s Hiroshima bombing and Chernobyl nuclear power plant disaster [24, 25].

In 2007, the International Commission on Radiological Protection issued recommendations that named the ovaries and testes, bone marrow, and eye lens as the most radiosensitive organs [26]. In this context, our students showed good knowledge as 65.1% of them identified ‘the testis and ovaries’ as the most sensitive organs. Other studies also showed abundant knowledge in radiosensitive organs among medical students. O’Sullivan et al. [23] indicated that 51% of the medical students were aware that the kidney is less sensitive to radiation compared with gonads. Hamarsheh and Amro [27] conducted an important study that assessed the knowledge and awareness of Palestinian radio technologists regarding radiation hazards, and they found that 6.9% named the lungs, 4.9% named the stomach, and 2.5% named the gonads as the most radiosensitive organs.

About 42% of our sample successfully identified ultrasound as the safest imaging method. Surprisingly, a considerable number of participants incorrectly named X-ray, CT, or MRI as the safest imaging approach which revealed an existing gap in the knowledge of Syrian medical students regarding imaging-associated hazards. As CT and MRI are only requested when indicated, we assessed our sample’s knowledge about the most common contraindications for these two imaging techniques. Regarding CT contraindications, 65.4% of our participants chose allergy to radio-contrast agents, 56.9% chose pregnancy, 42.8% chose renal failure, and 14.1% chose liver failure. However, only 8.2% responded with all four correct answers. As for MRI contraindications, the presence of metal foreign bodies, pacemaker, and claustrophobia was selected by 72.9%, 46.8%, and 43.5% of our students, respectively. Again, only a small percentage (21.6%) responded with all four correct answers. This gap in knowledge was also observed in an American study that was conducted by Prezzia et al. [21]. These authors reported on the opinion and knowledge of American fourth-year medical students regarding radiology, and they found that only 26.4% of the participants answered with all the correct answers. Regarding radiation doses, 78.4% of our respondents were not aware of the annual limit for X-ray imaging. Another study that reported lack of knowledge in radiation dosage was that conducted by Sundaran Kada. The author observed that 89% of the Norwegian medical students did not know that there are no limits on MRI doses given to a patient as long as it is medically justified [20]. The risks associated with ionizing radiation can be managed by using the lowest dose known to achieve the required image quality [28]. Implementing such measures allows for an unlimited number of X-ray images per year while ensuring the safety of the patient.

The uncontrolled use of procedures that employ ionizing radiation for body imaging has raised concerns about cancer risks [29, 30]. In our study, 33.5% of the students correctly placed the chance of a 30-year-old woman developing cancer after undergoing CT of the abdomen at 1 in 600. In contrast, Prezzia et al. [21] indicated that only 8.6% of their sample responded correctly to this question. Finally, O’Sullivan et al. [23] reported a high level of knowledge among their participants as 70% of the medical students were aware of the association between CT and increased cancer risk. In order to minimize the risks associated with the use of ionizing radiation, any physician who orders radiological imaging must have enough clinical experience and specific knowledge about these investigations and their possible risks on targeted groups. In our study, 51.3% and 27.9% of the students correctly recognized that US and MRI, respectively, do not involve ionizing radiation. In contrast, by O’Sullivan et al. showed better results as only 4.6% and 16.4% of their sample incorrectly associated US and MRI, respectively, with ionizing radiation [23]. The misconception among our students carried on to the estimation of CT and PET-CT radiation dosages, as only a small number of students knew that chest CT and full body PET-CT expose the patient to radiation levels that are equivalent to 100–500 chest X-rays (16.7%) and > 500 chest X-rays (26.4%), respectively. In their study of the awareness about ionizing radiation exposure among Australian senior medical students and interns, Zhou et al. reported comparable results where only 11.1% and 21.7% of their participants correctly estimated the ionizing radiation doses of abdominal CT and PET scan, respectively [5]. Moreover, 73.6% of the participants in our study underestimated PET-CT radiation dose and 18.3% dismissed that CT employs ionizing radiation. Other studies have also reported misconception in this field. Sundaran Kada noted that 1% of their participants thought that CT of the abdomen does not employ ionizing radiation. Similarly, Faggioni et al., who assessed awareness of radiation protection and dose levels of imaging procedures among Italian medical students, radiography students, and radiology residents, observed that 1.8% of medical students answered that CT involves no ionizing radiation. Moreover, they indicated that 5.5% of medical students did not associate PET-CT with radiation exposure [20, 31]. In our study, the misconceptions about radiation doses can be attributed two main factors. The first is lack of information provided by lectures, especially since only 24.4% of the students considered lectures as their source of information, and the second is unavailability of preclinical radiology rotations. This lack of knowledge has to be addressed in the medical education programs in Syria. Otherwise, doctors will continue to unknowingly expose patients to radiation from unnecessary imaging which will increase their risk for developing cancer [20].

Training in radiology in the first year of medical school is a necessity, especially for those interested in the field as a future career [32, 33]. Our results showed that 73% of the students did not complete a radiology rotation. Other studies supported our findings. Muzumdar et al. reported that only 35% of English medical students completed a rotation in IR. Similarly, Alnajjar et al. observed that only 25% of Saudi medical students completed or were planning to complete an elective in IR. Finally, Agrawal et al. noted an alarmingly low rate of IR rotation completion at 5.7% among Indian medical students [3, 34, 35]. Students who had a previous rotation in IR tended to be more informed about the specialty [3]. This shows that mandatory radiology exposure in undergraduate years is crucial, because the majority of specialties within the hospital refer to the radiology department. Interestingly, we were only able to show a slight difference in knowledge scores between students who completed a radiology rotation and those who did not. This is indicative of a potential gap in radiology training programs in Syria that needs to be addressed. We must ensure that radiology rotations continue to fulfill their intended purpose of educating medical students in this field.

In our study, only 24.5% of the respondents considered specializing in radiology for their future career. This result indicates a significantly low interest in radiology among Syrian medical students. Various studies from different parts of the world showed similar findings [3, 22, 36], which indicates that low interest in radiology is a global phenomenon. The primary reasons that our students gave for dismissing radiology were fear of radiation exposure and (23.3%) and a general lack of interest in this field (21.2%). To begin with, Syrian has legislations and guidelines on the proper use of ionizing radiation [37]. Therefore, the fear of radiation exposure can only be justified by the lack of knowledge of the implemented radiology safety precautions in Syria. Recent studies have made similar observations as they reported low levels awareness regarding essential radiology protection regulations [15, 38–43]. For instance, Alreshidi et al. [41] assessed the level of knowledge of Saudi medical students in radiology and found that only 11% of the respondents considered themselves adequately informed about radiology protection measures. On the other hand, lack of interest in radiology can be attributed to lack of knowledge in this field. Lately, interventional radiology has shown a rapid growth in the medical field worldwide [35, 36, 44]. However, like China, America, and most European countries, IR is not officially recognized as a specialist subject by the Syrian government [45]. In our study, 78.8% of the students who had previously heard about IR showed an exquisite interest in the field and considered radiology as a future career (*χ*^2^ = 21.879, *p* = 0.000). Similarly, Ghattan et al. surveyed second-year American medical students for their opinions on IR before and after a 1-h case-based introductory lecture in IR, and they found that interest in IR as a career choice increased from 19% before the course to 33% after the course [44]. Moreover, Branstetter et al. [46] have shown that education on diagnostic radiology enhances personal opinions and increases the number of students who would consider radiology as a specialty. These findings emphasize the impact of knowledge in IR on the increased interest in radiology as a career choice.

Finally, our students chose the Internet (33.5%), social media (30.5%), and university lectures 24.2% as the top three sources of information on radiology. Similarly, Wang et al. [45] revealed that the Internet (43%), teachers and textbooks (37%), and newspapers and TV (26.5%) were some of the resources that Chinese medical students used to learn about radiology. In Syria, like many other countries, medical students are provided with resources about radiology via different modes. Efforts must be made to ensure that medical students have constant access to online journals and media outlets that provide information about radiology, such as electronic textbooks, medical databases, videos, and podcast material.

## Limitations

This study was conducted in one institution in Syria and may not represent the overall situation in the country, even though it provides a valuable insight into the subject. Moreover, our sample of medical students may not reflect the actual situation for overall Syrian medical students. There may be a need to conduct a similar study on a national level for better generalization. Finally, the study did not take into consideration the impact of the Syrian crisis on radiology knowledge, as we did not find any trusted published data on the effect of the Syrian on the education system.

## Conclusion

This study showed that medical students at the Syrian Private University have an adequate knowledge in radiology with few misconceptions and a low interest in radiology as a future career. The level of awareness of the medical student regarding radiology can influence their decision in choosing radiology as a specialty. Intervention on an educational level to recruit the brightest radiologists as teaching faculty, train them in educating medical students, and support them throughout this process is pivotal to ensure that students are receiving the necessary training in radiology. Further evaluation in methods of teaching, input from medical boards, curriculum advisors, and guidance from radiologists is required. This will attract the finest students to the field and produce doctors who are aware of the risks and effects of imaging as well as the correct indications of radiology scans, and who are appreciative of the contributions of radiologists.

## Supplementary information

**Additional file 1**. The Questionnaire used to conduct the study and assess students’ knowledge. (In English).

## Data Availability

All data related to this paper’s conclusion are available and stored by the authors. All data can be made available by the corresponding author on a reasonable request.

## Abbreviations

CT: Computed tomography
DEXA: Dual energy X-ray absorptiometry
GPA: Grade point average
HCW: Healthcare worker
IR: Interventional radiology
IRB: Institutional Review Board
MRI: Magnetic resonance imaging
PAARS: Pan Arab Association of Radiological Societies
PET: Positron emission tomography
PG: Postgraduate
SD: Standard deviations
SPSS: Statistical Package for Social Sciences
SPU: Syrian Private University
US: Ultrasonography
