# Supplementary material for: Assessing diagnostic radiology knowledge among Syrian medical undergraduates
Source: Insights Imaging. 2020 Nov 23;11:124. doi: 10.1186/s13244-020-00937-9 (PMC7683655; doi:10.1186/s13244-020-00937-9)
Supplement: Supplementary file 1 — Additional file 1. The Questionnaire used to conduct the study and assess students’ knowledge. (In English). [file 13244_2020_937_MOESM1_ESM.docx]

# ELECTRONIC SUPPLEMENTARY MATERIAL

#
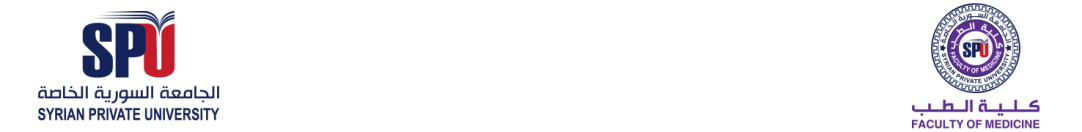
Appendix 1.

Ethical approval of the questionnaire was obtained from the Institutional Review Board (IRB), Faculty of Medicine.

| **Socio-demographic Characteristics** | |
| --- | --- |
| **Age:**  ⬜ Under 20 ⬜ 20-25 ⬜ Above 25 | **Mother's Education:**  ⬜Primary ⬜Secondary ⬜High School ⬜University ⬜Graduate |
| **Gender:**  ⬜Male ⬜Female | **Current residence:**  ⬜City ⬜Rural |
| **Social Status:**  ⬜Single ⬜In a relationship ⬜Married | **GPA:**  ⬜< 2.0 ⬜2.0 - 2.5 ⬜2.5 - 3.0 ⬜> 3.0 |
| **College Year:**  ⬜1st ⬜2nd ⬜3rd ⬜4th ⬜5th ⬜6th | |

| **Table 2. Background and experience n (269)** | | |
| --- | --- | --- |
|  | **yes** | **no** |
| Have you had a radiograph (of any kind) before? |  |  |
| Has any of your relatives had a radiograph (of any kind) before? |  |  |
| Are you interested in learning more about this field? |  |  |
| Have you completed a rotation in radiology? |  |  |
| Have you ever heard about interventional radiology\ before? |  |  |
| How would you rate your knowledge of radiology compared to other fields?  ◻️Poor ◻️Adequate ◻️Good ◻️Excellent | | |
| How would you rate your knowledge about the radiation dose of common radiological investigations?  ◻️Poor ◻️Adequate ◻️Good ◻️Excellent | | |
| How much of an impact does radiology have on the diagnosis process? ◻️minimal impact ◻️occasionally changes patient care ◻️ often changes patient care ◻️ as important as the physical exam ◻️ more important than a physical exam | | |

| **Table 3. Basic knowledge about radiology** | | | | | |
| --- | --- | --- | --- | --- | --- |
| What age group is the most sensitive to radiation? | **Children** | **teens** | **adults** | **elders** | **others** |
|  |  |  |  |  |  |
| Is there a specific number of radiographs that can be requested for the patient per year? | **yes** | **no** |  | | |
|  |  |  |  |  |  |
| Does radiation affect the fetus? |  |  |  |  |  |
| What type most affects the fetus? | **US** | **MRI** | **X-ray** | **CT** | **Others** |
|  |  |  |  |  |  |
| What is the safest radiological investigation? |  |  |  |  |  |
| What are the most sensitive organs to radiation? | **liver, bladder, kidney** | **lungs, colon** | **Breast** | **testis and ovaries** | **Others** |
|  |  |  |  |  |  |
| CT contraindications: | **allergy to radio contrast agent** | **renal failure** | **liver failure** | **pregnant women** | **Do not know** |
|  |  |  |  |  |  |
| MRI contraindications: | **pacemaker** | **metal foreign bodies** | **claustrophobia** | **Do not know** |  |
|  |  |  |  |  |  |
| What is your source of information? ◻️Internet ◻️social media ◻️lectures ◻️personal experience ◻️clinical rotations ◻️friends ◻️family ◻️TV ◻️magazines | | | | | |

| **Table 4. Levels of radiation exposure: (n=269)** | | | | | | |
| --- | --- | --- | --- | --- | --- | --- |
|  | Number of units equivalent to a chest X-ray (a chest X-ray = 1 unit) | | | | | |
| Procedure | **0** | **1-10** | **10-50** | **50-100** | **100-500** | **>500** |
| CT chest |  |  |  |  |  |  |
| MRI pelvis |  |  |  |  |  |  |
| PET-CT full body |  |  |  |  |  |  |
| US abdomen |  |  |  |  |  |  |
| What is the best estimate that a 30-year-old woman who undergoes a (CT) study of the abdomen and pelvis will develop cancer at some point in her life as a direct result of that imaging study? | | | **1/60** | **1/600** | **1/6000** | **1/60000** |
|  |  |  |  |  |  |  |

| **Table 5. radiology as a screening test** | | |
| --- | --- | --- |
|  | **yes** | **no** |
| We can perform a radiological screening test in (Mammography for breast cancer) |  |  |
| We can perform a radiological screening test in (US for abdominal aortic aneurysm) |  |  |
| We can perform a radiological screening test in (CT for lung cancer) |  |  |
| We can perform a radiological screening test in (DEXA for osteoporosis) |  |  |

| **Table 6. Radiology as a future career: (n=269)** | | | | | |
| --- | --- | --- | --- | --- | --- |
| How interesting is the subject matter in radiology? | it is worthless to me | it is dull but important | it is interesting only as it relates to other fields of medicine | it is interesting in its own | |
|  |  |  |  |  | |
| In your opinion, in general, a radiologist’s income for their services is compared with clinicians in medical and surgical specialties? | much less | a little less | the same | a little more | much more |
|  |  |  |  |  |  |
| How many years is radiology residency? | **3** | **4** | **5** | **6** |  |
|  |  |  |  |  |  |
| In your opinion, in general, a radiologist’s professional lifestyle is compared with clinicians in medical and surgical specialties? | much easier | slightly more  easier | the same | slightly more difficult | much more difficult |
|  |  |  |  |  |  |
| Would you consider a radiology specialty as a future career? | **yes** | **no** |  | | |
|  |  |  |  |  |  |
| If no, choose why?  ◻️lack of interest ◻️lack of knowledge ◻️lifestyle ◻️radiation exposure ◻ the community ◻️Others | | | | | |
